# Collagen-Enriched Immunomodulatory Hydrogel for Tendon Regeneration

**DOI:** 10.3390/gels12040317

**Published:** 2026-04-08

**Authors:** Shivam Patel, Jeremy Pan, An Phong Nguyen, Nahid Howard, Finosh G. Thankam

**Affiliations:** Department of Translational Research, College of Osteopathic Medicine of the Pacific, Western University of Health Sciences, Pomona, CA 91766, USA; shivam.patel1@westernu.edu (S.P.); jeremy.pan@westernu.edu (J.P.); anphong.nguyen@westernu.edu (A.P.N.); nahid.howard@westernu.edu (N.H.)

**Keywords:** rotator cuff tendon injury, immunocompatibility, antioxidant hydrogels, collagen-based scaffolds, TREM1-driven inflammation

## Abstract

Rotator cuff tendon injury (RCTI) is aggravated by the pro-inflammatory milieu elicited by TLR4 and TREM1 signaling. Hence, tendon tissue engineering approaches require considerations that address these inflammatory episodes to benefit active regenerative responses. The objective of this study was to engineer and evaluate the immunocompatibility of a tendon-mimetic hydrogel composed of a chitosan–polyvinyl alcohol (PVA) blend incorporated with Collagen-I and to assess LR12 delivery for addressing TREM1-driven inflammation in RCTI management. A chitosan–PVA-HEMA-Acrylic acid (CPHA) hydrogel was synthesized by blending the linear natural polysaccharide chitosan and linear synthetic polymer PVA in an aqueous phase, followed by incorporation and redox chain growth with HEMA using acrylic acid (AA). Interpenetration of Collagen-I in CPHA yielded the CPHA-C hydrogel. CPHA and CPHA-C hydrogels displayed ample surface functional moieties provided by the co-polymers, exhibited excellent porosity as revealed by SEM imaging (28.65 ± 6.85 and 41.56 ± 18.00, respectively, for CPHA and CPHA-C), and were amphiphilic, as evident by contact angle analysis (~70 for CPHA and CPHA-C). Both hydrogels displayed a progressive release profile for the TREM1-inhibitory peptide LR12 for 7 days, whereas the LR12-loaded CPHA hydrogel exhibited increased TREM1 inhibition in LPS-challenged RAW264.7 macrophages. CPHA and CPHA-C hydrogels were immunocompatible and masked the oxidative damage in RAW264.7 macrophages, as evident by decreased levels of mitochondrial superoxide and ROS. Additionally, the CPHA hydrogel displayed an increased TGFβ/TLR4 ratio (0.24), whereas the CPHA-C (−0.52) system showed a decreased ratio upon exposure to tenocytes and macrophages. Overall, the findings highlight the potential of CPHA and CPHA-C hydrogels as candidates for tendon regenerative applications.

## 1. Introduction

Rotator cuff tendon injury (RCTI) is a major musculoskeletal complaint in the United States affecting around 20.7% of the population [1], with a more than 200% increase in repair surgeries for RCTI, indicating a significant rise in incidence [2]. Notably, the advanced-age population is highly susceptible to RCTI due to overuse leading to increased degenerative changes in the tendon [3]. Conventional management interventions are often limited by the persistence of pain and inflammation, fatty infiltration, tendon matrix disorganization, and incomplete restoration of structural and functional integrity resulting in poor clinical outcomes [4,5]. Hence, tendon replacement strategies including tissue engineering have emerged to address these challenges, aiming to accelerate the functional recovery of the tendon following RCTI [6,7]. Among these strategies, hydrogel-based biomaterials have shown promise for in vitro tendon tissue engineering due to their physicochemical resemblance to the native tendon matrix [8].

Persistent inflammation aggravates RCTI, which creates significant challenges for the effectiveness of conventional tissue engineering approaches [9]. Unfortunately, tendon-mimetic hydrogel templates that simultaneously support regenerative and inflammatory responses remain rare. Previous research from our group has identified the role of triggering receptors associated with myeloid cells 1 (TREM1) in exacerbating tendon inflammation in human patients and animal models [9,10]. The exacerbation of inflammation via TREM-1 hurdles regeneration/repair of the tendon following RCTI [11]. Therefore, targeting TREM1 has emerged as a promising strategy for the management of RCTI in the pre-clinical setting [12]. The inhibition of TREM1 has been achieved through binding using LR12, a dodecapeptide (LQEEDAGEYGCM) that prevents the activation of the adaptor protein DNAX-activating protein-12 (DAP-12) [13]. LR12 has previously been utilized to target inflammation in multiple diseases [14,15,16,17]; however, information regarding the application of LR12 in tendon regeneration, specifically via hydrogel delivery systems, remains scarce. We and others have reported that the decreased ratio of Collagen-I to Collagen-III (Collagen-I:III ratio) in the tendon matrix is a major pathological outcome resulting in impaired tendon biomechanics and delayed healing, subsequently resulting in RCTI [18,19]. Hence, hydrogel templates intending to reinstate tendon function following RCTI warrant careful consideration to restore the proper Collagen-I:III ratio. Additionally, designing scaffolds that home shoulder tenocytes and mimic the native tendon (comprising ~70% Collagen-I in the dry weight) has been a viable strategy [20]. For instance, the incorporation of bovine Collagen-I into a chitosan-based hydrogel has significantly accelerated tendon regeneration, restored the mechanical properties of the tendon matrix, and facilitated the proliferation and migration of loaded tenocytes [21]. Therefore, hydrogel templates designed to simultaneously address Collagen-I depletion, facilitate tenocyte replenishment, and target TREM1 hold strong potential to enhance tendon regeneration following RCTI. Chitosan–PVA and collagen-based hydrogels have been employed for wound healing [22,23,24]; however, their application for tendon regeneration is limited. Additionally, immunomodulation by specifically targeting TREM1 using chitosan-based hydrogels remains unexplored for RCTI management. Hydrogel-based strategies to restore the Collagen I-to-III ratio are critical for enhancing tendon healing but remain understudied [25]. Hence, tendon-inspired hydrogels comprising Collagen-I, the native tendon matrix component, are highly desirable for RCTI management [6]. Despite the elusive relationship between Collagen-I expression and TREM1 signaling in RCTI, approaches that simultaneously promote Collagen-I restoration and regulate TREM1 signaling demonstrate immense clinical significance. However, such combinatorial strategies remain unavailable in the current literature. Hence, our objective is to engineer a tendon-mimetic hydrogel composed of a chitosan–PVA blend integrated with Collagen-I and to evaluate its immunocompatibility and capacity for LR12 delivery to target TREM1-driven inflammation for RCTI management.

## 2. Results and Discussion

### 2.1. Synthesis of CPHA and CPHA-C Hydrogels

**Figure 1:**
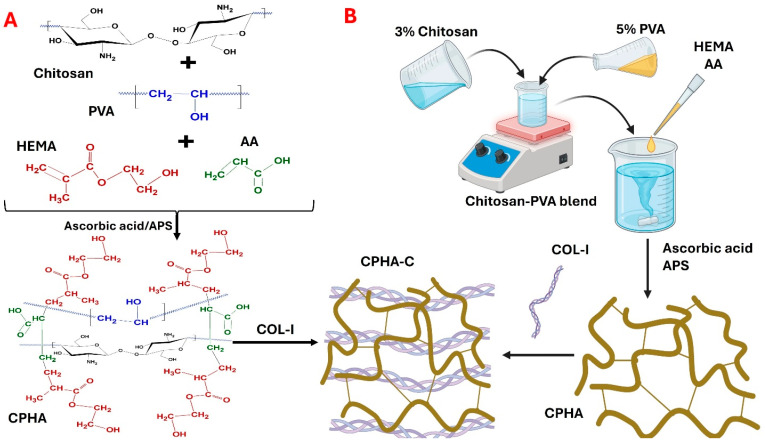
(**A**) The proposed co-polymer structure. This is a proposed network model and not a structurally confirmed architecture. (**B**) A schema of the synthesis of CPHA and CPHA-C hydrogels.

The CPHA hydrogel was synthesized by blending the linear natural polysaccharide chitosan and linear synthetic polymer PVA in an aqueous phase followed by the incorporation and redox crosslinking of HEMA using AA. CPHA hydrogels adopted semi-IPN chemistry, favored by the redox catalyzed chain growth polymerization of HEMA-AA systems. Additionally, the H-bonds utilizing the O atoms of various functional groups and nitrogen from -NH_2_ of chitosan stabilize the semi-IPN. The interpenetration of Collagen-I to CPHA to impart tendon-mimetic features resulted in the formation of CPHA-C (Figure 1). Moreover, both hydrogels were stable, flexible, and able to be handled using tweezers without breaking. Both hydrogels retained their shape and integrity in water, PBS, and DMEM.

### 2.2. Physiochemical Features of CPHA and CPHA-C Hydrogels

**Figure 2:**
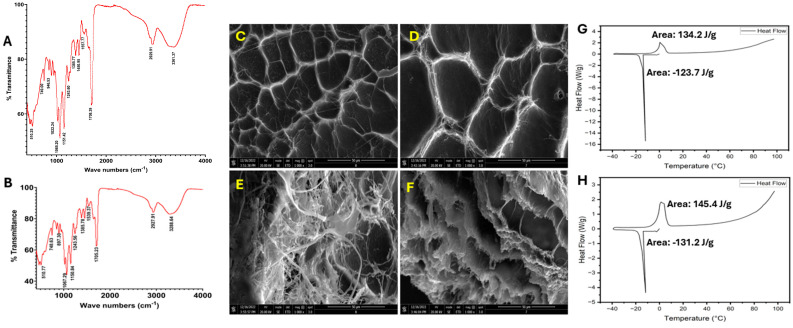
AT-IR spectra of freeze-dried (**A**) CPHA and (**B**) CPHA-C hydrogels reveal the characteristic surface functional groups. SEM imaging reveals the (**C**) surface and (**E**) inner porous architecture of the CPHA hydrogel and (**D**) the surface and (**F**) inner porous architecture of the CPHA-C hydrogel. A DSC thermogram of (**G**) CPHA and (**H**) CPHA-C hydrogels showing the melting of freezing water.

AT-IR spectrum analysis (Figure 2A,B) of the CPHA and CPHA-C hydrogels revealed similar spectra with a broad peak around 3250 cm^−1^, suggesting the presence of –OH groups contained in chitosan and PVA. The peak around 2900 cm^−1^ represents -OH stretching vibrations, suggesting free carboxylic acid groups. The sharp peak around 1700 cm^−1^ represents carbonyl stretching, suggesting the presence of ester linkages and carboxylic acid groups. The peak around 1550 cm^−1^ represents alkene C=C stretching. The peak around 1450 cm^−1^ displays the –OH bending of PVA. The sharp peak around 1020–1060 cm^−1^ reflects C–O–C stretching of the glycoside linkages in chitosan and PVA. The minor peak around 840 cm^−1^ reflects C–H bending in chitosan and PVA. Also, the I, II, and III bands of collagen are represented by peaks around 1650, 1540, and 1240 cm^−1^ in CPHA-C hydrogels. Overall, the IR spectral analysis suggests the presence of ample functional groups possibly due to the respective fragments of chitosan, PVA, HEMA, and collagen on the surface of the hydrogels.

Both CPHA and CPHA-C hydrogels displayed well-oriented pore architecture on their surface, as evident by SEM imaging (Figure 2C,D). CPHA displayed decreased pore length compared to CPHA-C; however, the difference was not statistically significant (Table 1). Additionally, the SEM images of the cross-sectional area displayed well defined pore interconnectivity (Figure 2E,F). The advancing angles observed for CPHA and CPHA-C were 71.49 ± 2.91° and 68.81 ± 2.25°, respectively. The receding angles observed for CPHA and CPHA-C were 73.04 ± 2.12° and 68.84 ± 2.67°, respectively. The alteration in advancing and receding contact angles between CPHA and CPHA-C hydrogels was not statistically significant (*p* = 0.7751) (Table 1).

**Figure 3:**
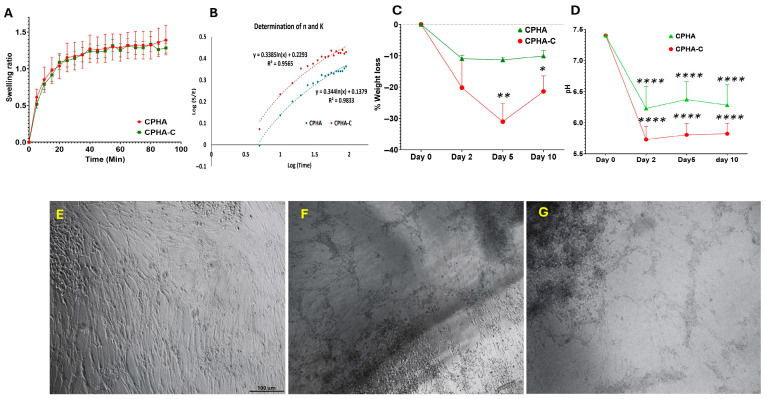
(**A**) A graph showing the progressive swelling ratio of CPHA and CPHA-C hydrogels (mean ± SEM). (**B**) A representative graph (n = 5) for the determination of the diffusional exponent (n) and the swelling constant (k) for CPHA and CPHA-C hydrogels from the plot of log(S/E) along the Y-axis and log(time) along the X-axis. Biodegradation of CPHA and CPHA-C hydrogels showing the decline in (**C**) dry weight and (**D**) pH upon aging in PBS for 10 days. The direct contact assay of swine tenocytes shows the absence of changes in cell morphology in the vicinity of CPHA scaffolds (**F**) and CPHA-C (**G**) hydrogels compared with control (**E**). Images were taken at 20× magnification under a phase-contrast microscope (* *p* < 0.05; ** *p* < 0.01; **** *p* < 0.0001).

Swelling was more elevated in CPHA-C than CPHA; however, it was not statistically significant (*p* = 0.2900) (Table 1). Also, similar levels of EWC were displayed by both hydrogels (*p* = 0.7308) (Table 1). The DSC thermogram of both CPHA and CPHA-C hydrogels showed sharp peaks around 0 °C, corresponding to the melting of frozen water observed in the heating curve. The melting enthalpy of CPHA and CPHA-C hydrogels was 134.2 J/g and 145.4 J/g, respectively (Figure 2G,H). The freezing water content (Wf) was calculated from the enthalpy of the melting of pure water (334 J/g), and the non-freezing bound water (Wnb) was calculated from the difference between EWC and Wf [26]. Wf values were 39.58% and 43.53% and Wnb values were 17.59% and 12.51%, respectively, for CPHA and CPHA-C hydrogels (Table 1). CPHA displayed increased Wnb compared to CPHA-C, whereas Wf was higher in CPHA-C. Both CPHA and CPHA-C hydrogels displayed progressive swelling curves under sink conditions that stabilized after 90 min (Figure 3A). The swelling kinetics were quantified based on water intake at 5 min intervals, and a standard curve was employed to calculate the ESR (E), swelling ratio (S), swelling constant (K) and diffusional exponent (n) (Figure 3B) (Table 1). K and n were derived, respectively, by the intercept and slope from the plot of log(S/E) vs. log(time). The diffusional exponent (n) was 0.3447 for CPHA and 0.4051 for CPHA-C, whereas the swelling constants were 0.1484 and 0.0848, respectively. The number of TWAS for CPHA was 9.24 × 10^20^ ± 6.32 × 10^19^ and 9.87 × 10^20^ ± 2.14 × 10^20^ for CPHA-C; however, the difference in TWASs between CPHA and CPHA-C hydrogels was not statistically significant (*p* = 0.6905) (Table 1).

### 2.3. CPHA and CPHA-C Hydrogels Are Biodegradable

Both CPHA and CPHA-C hydrogels displayed a progressive loss of dry weight compared to the initial dry weight. CPHA-C displayed increased degradation compared to CPHA; however, this was not statistically significant (Figure 3D). Similarly, both hydrogels exhibited a significant decline in pH compared to the control. Notably, the pH drop was greater for CPHA-C compared to CPHA (*p* = 0.0167, on day 10) (Figure 3E).

### 2.4. CPHA and CPHA-C Hydrogels Supported the Metabolic Stability of Tenocytes

**Figure 4:**
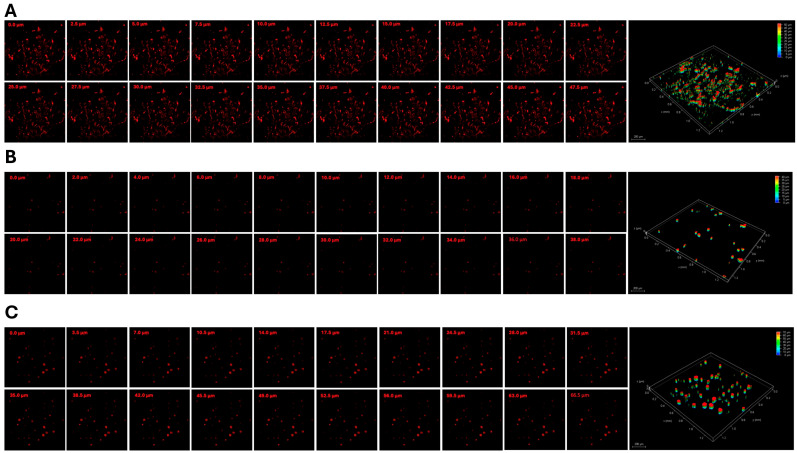
The gallery of Z-stack images displaying the infiltration of rhodamine tenocyte cells toward the interior of CPHA scaffolds (**B**) and CPHA-C scaffolds (**C**) stained for 5 days after seeding compared to the (**A**) control. The right panel shows the three-dimensional rendering obtained through the overlay of Z-stacks, visualizing the penetration depth of the tenocytes. Images were taken at 20× magnification under a Leica thunder scanning microscope.

The swine shoulder tenocytes grown on the surface of CPHA and CPHA-C hydrogels displayed the absence of alterations in their morphology or cell death. Contact with the hydrogels failed to arrest the proliferative capacity of tenocytes, similar to the control (Figure 3F–G). Additionally, the tenocytes grown on the media extracted from CPHA and CPHA-C hydrogels displayed metabolic activity greater than 90%, suggesting the absence of toxic degradation products. Both CPHA and CPHA-C hydrogels displayed similar levels of viability (Table 1). Cell survival predominated in both hydrogels, comparable to the control. Rhodamine–phalloidin staining displayed the increased penetration of tenocytes onto the interstices of the CPHA-C hydrogel (~70 µm) compared to the CPHA system (~40 µm). Also, greater colonization of cells was observed in the CPHA-C hydrogel (Figure 4).

### 2.5. CPHA and CPHA-C Hydrogels Maintained Oxidative Balance

**Figure 5:**
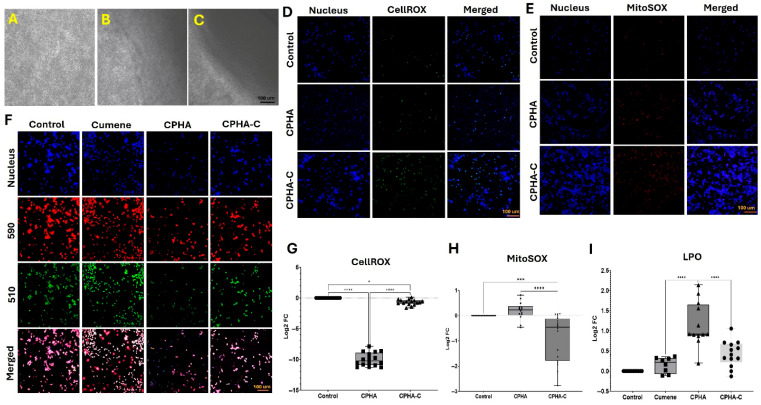
The direct contact assay of RAW264.7 macrophages showing the absence of changes in cell morphology in the vicinity of CPHA scaffolds (**B**) and CPHA-C (**C**) hydrogels compared with control (**A**). Images were taken at 20× magnification under a phase-contrast microscope. Representative images for the (**D**) CellROX and (**E**) MitoSOX assays displaying the decreased activities in CPHA and CPHA-C hydrogels and (**G**,**H**) the quantification of MFI, respectively. Images in the left column show nuclear staining with DAPI, images in the middle column show the level of CellROX/MitoSOX, and images in the right column display the overlay of CellROX/MitoSOX staining with DAPI. Representative images for the (**F**) lipid peroxidation assay exhibiting fluorescence at 590 nm and 510 nm in cumene, CPHA and CPHA-C hydrogel-treated RAW264.7 cells, and (**I**) quantification of the ratio of MFI from 590 nm and 510 nm. The graphs represent log_2_ fold change calculated from MFI values normalized to the controls (* *p* < 0.05; *** *p* < 0.001; **** *p* < 0.0001).

RAW264.7 macrophages cultured in direct contact with CPHA and CPHA-C hydrogels displayed a morphology comparable to the controls, suggesting the absence of macrophage activation. Additionally, neither of the hydrogels impaired macrophage growth or proliferation (Figure 5A–C). Both CPHA (*p* < 0.0001) and CPHA-C (*p* = 0.0157) significantly reduced intracellular reactive oxygen species (ROS) levels compared to the control, with CPHA showing greater ROS suppression than CPHA-C (*p* < 0.0001) (Figure 5D,G). Also, CPHA-C significantly reduced mitochondrial superoxide levels relative to both the control (*p* = 0.0022) and CPHA (*p* = 0.0003), whereas CPHA showed no significant difference from the control (*p* = 0.7741) (Figure 5E,H). Lipid peroxidation (LPO), assessed by the 590/510 fluorescence ratio, was significantly elevated in the cumene hydroperoxide control compared to both CPHA (*p* < 0.0001) and CPHA-C. However, the reduction in LPO observed with CPHA-C was not statistically significant (*p* = 0.3098). Overall, CPHA hydrogels demonstrated superior antioxidant and anti-inflammatory effects compared to CPHA-C (*p* < 0.0001) (Figure 5F,I).

### 2.6. CPHA Hydrogels Maintained TGFβ/TLR4 Ratio

**Figure 6:**
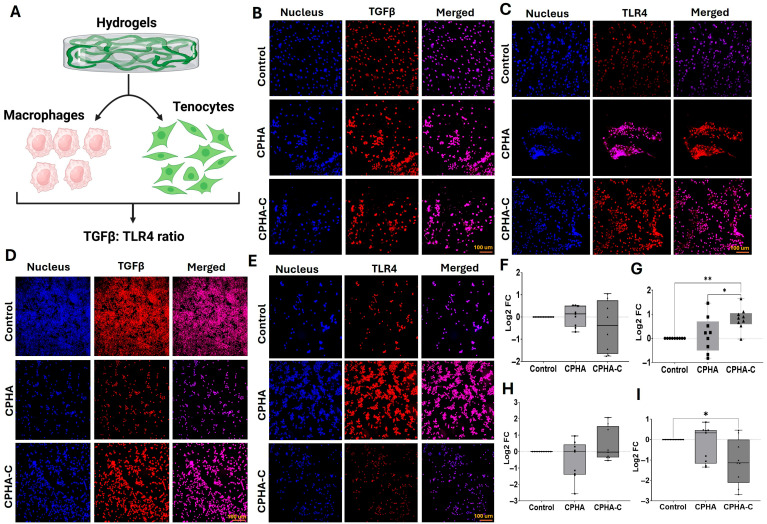
(**A**) An overall schema of experiments. Representative images showing the immunofluorescence analysis for TGFβ expression in (**B**) tenocytes and (**D**) RAW264.7 macrophages and TLR4 expression in (**C**) tenocytes and (**E**) RAW264.7 macrophages. Images in the left column display nuclear staining with DAPI; images in the middle column represent the expression of TGFβ/TLR4 and the right column shows an overlay of TGFβ/TLR4 staining with DAPI. Images were acquired at 20× magnification using a Leica Thunder microscope. The quantification of protein expression represented as log_2_ fold change calculated from MFI normalized to the control. (**F**,**G**) The quantification of TGFβ and TLR4, respectively, in tenocytes. (**H**,**I**) The quantification of TGFβ and TLR4, respectively, in macrophages (* *p* < 0.05; ** *p* < 0.01).

Swine shoulder tenocytes and RAW 264.7 macrophages were exposed to CPHA and CPHA-C hydrogels, and expression levels of TGFβ and TLR4 were quantified (Figure 6A). In tenocytes, TGFβ expression was reduced following interaction with CPHA-C hydrogels; however, this reduction was not statistically significant compared to either the control (*p* = 0.4273) or CPHA group (*p* = 0.3661). No significant difference was observed between the CPHA and control groups (*p* = 0.4273) (Figure 6B,F). In contrast, TLR4 expression was significantly elevated in tenocytes treated with CPHA-C compared to both the control (*p* = 0.0054) and CPHA groups (*p* = 0.0273) (Figure 6C,G). Notably, the TGFβ/TLR4 ratio in tenocytes was 0.24 for CPHA and –0.52 for CPHA-C-treated in raw 264.7 macrophages; TGFβ expression showed a non-significant decrease with CPHA treatment (*p* = 0.4466) and a non-significant increase with CPHA-C treatment (*p* = 0.5928) compared to control (Figure 6D,H). TLR4 expression was reduced in both CPHA (*p* = 0.8420) and CPHA-C (*p* = 0.0254)-treated macrophages, with only the CPHA-C group showing a statistically significant decrease. The downregulation of TLR4 in CPHA-C was greater than that in CPHA; however, it was not statistically significant (*p* = 0.0832) (Figure 6E,I). Interestingly, the TGFβ/TLR4 ratio in macrophages was 2.38 for CPHA and –0.38 for CPHA-C treatment. The negative values indicate that the ratio is below the control which has a normalized log_2_ value of zero.

### 2.7. LR-12-Loaded CPHA Hydrogel Displayed TREM1 Inhibition

**Figure 7:**
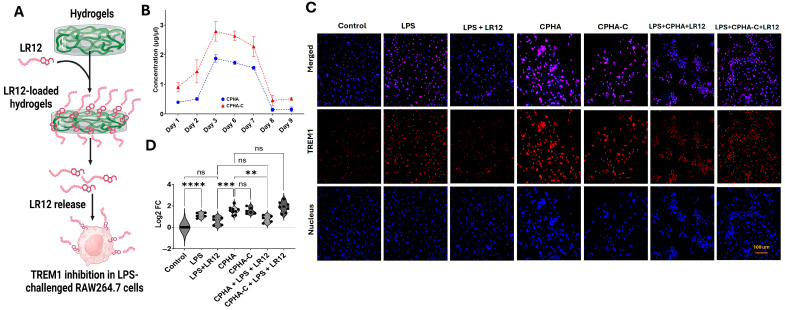
(**A**) An overall schema of experiments. (**B**) LR12 release profile of CPHA and CPHA-C hydrogels quantified for 9 days (n = 3). (**C**) Representative images showing the immunofluorescence analysis for TREM1 expression in LPS-challenged RAW264.7 macrophages upon treatment with LR12-loaded hydrogels. Images in the bottom row display nuclear staining with DAPI, images in the middle row represent the expression of TREM1, and the upper row shows an overlay of TREM1 staining with DAPI. Images were acquired at 20× magnification using a Leica Thunder microscope. (**D**) The quantification of TREM1 expression represented as log_2_ fold change calculated from MFI normalized to control (** *p* < 0.01; *** *p* < 0.001; **** *p* < 0.0001; unlabeled values are not significant (ns)).

LR12 was incorporated into CPHA and CPHA-C hydrogels to evaluate its release profile and TREM1-inhibitory effects (Figure 7A). Both hydrogels exhibited a similar release pattern, with a progressive increase in LR12 release over the first 3 days, sustained release through day 7, and a return to baseline levels by day 9. Notably, CPHA-C demonstrated a higher cumulative release compared to CPHA (Figure 7B). Lipopolysaccharide (LPS) stimulation significantly upregulated TREM1 expression in RAW 264.7 macrophages compared to the untreated control (*p* < 0.0001). This upregulation was markedly suppressed by direct LR12 treatment (*p* = 0.0585). The interaction of CPHA (*p* < 0.0001) and CPHA-C (*p* < 0.0001) hydrogels with RAW 264.7 cells significantly increased TREM1 expression. Interestingly, CPHA hydrogels loaded with LR12 showed a reduction in TREM1 expression in LPS-challenged macrophages similar to the LPS-LR12 control (*p* = 0.9680). Interestingly, TREM1 expression in CPHA-C hydrogels loaded with LR12 remained unchanged in LPS-stimulated macrophages. Furthermore, TREM1 expression in the CPHA group was significantly lower than that in the CPHA-C group (*p* < 0.0001) (Figure 7C,D).

### 2.8. Discussion

Hydrogels have been found to have potent biocompatibility and biodegradability, making them ideal candidates for creating an extracellular matrix (ECM)-like microenvironment in regenerative applications across various tissues [27,28,29,30,31]. Biocompatibility of regenerative hydrogels is critical to preventing foreign body reactions that can impair the healing process. This study focuses on evaluating the immunocompatibility and functional performance of hydrogels synthesized from the linear natural polysaccharide chitosan and the linear synthetic polymer PVA crosslinked with a HEMA-AA redox system for tendon regeneration. Following RCTI, the physiological healing process progresses through three key phases: inflammation, proliferation, and remodeling; however, prolonged inflammation exacerbates tissue damage and impair recovery [5,32]. Notably, the inflammatory response in RCTI has been closely linked to the upregulation of TREM-1 and other pro-inflammatory markers that disrupt appropriate healing [9,10,33]. During the remodeling phase, these newly deposited collagen fibers are organized and aligned, restoring the structural and functional integrity of the tendon [34]. Therefore, scaffold designs that support collagen deposition and tenocyte activity offer translational benefits for improving tendon repair outcomes.

Our investigations have developed a novel tendon-mimetic CPHA hydrogel system using the biocompatible polymers chitosan and PVA embedded with bovine COL-I. Notably, the successful and safe clinical applications of bovine COL-I have been reported for human RCTI management, exhibiting minimal immunological risk [35]. Hence, the incorporation of collagen represents a scalable strategy to ensure biocompatibility. The natural polysaccharide chitosan has received growing interest in tendon tissue engineering due to its non-toxic and biocompatible properties which are comparable to human physiology [36]. Chitosan-based hydrogels displayed tenocyte alignment that deposited organized collagen fibrils, accelerating tendon regeneration by upregulating reparative genes [37]. Similarly, the FDA-approved synthetic polymer PVA offers significant mechanical strength and flexibility within implants which could overcome the mechanical challenges associated with chitosan-based hydrogels [38]. Moreover, the rapid disintegration of PVA due to its extreme hydrophilicity [26] was effectively controlled by interpenetration with chitosan and crosslinking using a HEMA-AA redox system. Furthermore, the CPHA hybrid system has benefitted from the thermal stability, mechanical properties, and antimicrobial activity provided by the co-polymers employed for synthesis [39,40,41].

The water content and water-holding capacity of hydrogels are crucial for tendon regeneration as they directly impact the exchange of nutrients and fluid essential for cell survival [42]. This substantial swelling pattern facilitates the ability of these hydrogels to create and maintain a hydrated environment, which is essential for the survival and proliferation of tenocytes [43,44]. Considering the bioengineering of hypovascular tissues such as tendons, appropriate hydration status of the scaffolds is critical for nutrient diffusion, waste removal, and mechanical cushioning, thereby contributing to enhanced cell viability [6,45]. Additionally, the porosity exhibited by CPHA and CPHA-C allows for the retention of water molecules within their networks. Generally, the cells used in tendon regeneration applications are largely anchorage-dependent, which emphasizes the importance of uniform porosity for the successful distribution, adhesion, growth, proliferation, and migration of seeded cells to promote proper tendon regeneration [27].

In addition, the hydrophilic nature of the hydrogels, assessed through their wettability using dynamic contact angles, contributes to their water-holding capacity [46]. The contact angle measurements of CPHA and CPHA-C hydrogels demonstrated a more hydrophilic nature, indicating a strong affinity for water. Such hydrophilic hydrogels with partial hydrophobic characteristics promote protein adsorption and cell adhesion, which are critical for tenocyte attachment and function. The lower contact angle in CPHA-C has been attributed to the inherent hydrophilicity of collagen, which possesses additional hydrophilic functional groups such as amines, hydroxyl, and carboxyl groups along its polypeptide chain [47,48]. Generally, collagen-based hydrogels exhibit a porous and fibrous network, allowing more water molecules to distribute evenly across the surface and subsequently lowering the contact angle [49]. Furthermore, similar advancing and receding contact angles in both hydrogels are correlated with stable wetting features that support long-term moisture retention and cellular compatibility [50]. Supporting this finding, SEM imaging of CHPA-C revealed a larger and extensive network of heterogeneous porous voids compared to CPHA, as evident by the higher SD values. This has been attributed to the increased freezing water content that results in water crystals of varied size during freeze-drying depending on the local surface chemistry [51].

The surface chemistry of biomaterials plays a crucial role in regulating the local inflammatory responses through macrophage activation. The contact of biomaterials with macrophages activates inflammatory mediators including TLRs and TREM1 and the extent of their expression determines the success of the implant and host response [52,53]. Interestingly, biomaterials rich in surface -COOH and -NH2 trigger transient TLR4 expression with a concomitant upregulation of downstream anti-inflammatory genes including IL-1RA and IL-10 [52]. Hence, the -COOH and -NH2 moieties provided by chitosan and other components of CPHA hydrogels promote immunomodulation. As the hydrophobicity of biomaterials influences immunomodulation [54], the surface hydrophobicity, evident from the contact angle values, of CPHA hydrogels favors immunocompatibility. Similarly, the formation of protein corona is influenced by the surface chemistry and hydrophobicity, which directs macrophage polarization, where albumin-rich corona promotes immunomodulation [55]. Similarly, the fragments of unreacted HEMA and AA were left unreacted in the hydrogel formulation, allowing for the use of redox chemistry with their double bonds for scavenging reactive oxygen species [56]. However, the characterization of such unreacted fragments and their potential benefits warrant further investigations. Interestingly, both hydrogels effectively shielded ROS production in RAW264.7 cells, supporting this assumption. However, further optimizations are warranted for determining the free radical scavenging potential of CPHA hydrogels. Overall, CPHA hydrogels provide appreciable physiochemical cues that facilitate immunocompatibility, suggesting their potential in RCTI management.

The water content and water-holding capability of the CPHA hydrogels are controlled by Fickian diffusion, as evident by the diffusional exponent (n) which was less than 0.5 m. The Fickian diffusion model of these hydrogels was driven by the quantity of water per unit area in unit time which is proportional to TWASs [26]. The relatively higher TWASs displayed by both hydrogels facilitated the increased water intake, attaining maximum swelling within 90 min, as reflected in the swelling studies. Moreover, the freezing free and bound water content in CPHA and CPHA-C hydrogels influence the overall water content and diffusion potential [51]. Additionally, hydrophilicity determines the inflow of water molecules, which is related to TWASs. Hence, the water content and the nature of diffusion in CPHA and CPHA-C hydrogels ensure the loading–release kinetics of cells/compounds onto their interstices, which is essential for successful tendon tissue engineering. However, further detailed investigations are warranted for optimizing such cellular responses with respect to the rate of tendon healing.

Usually, hydrogels are susceptible to natural degradation post implantation in vivo while integrating with the host tissue [57]. The loss of dry weight of CPHA and CPHA-C reflects the natural erosion of the polymer network in physiological conditions. The increased rate of degradation observed for CPHA-C has been attributed to the biophysical properties of collagen, which has an increased susceptibility to both enzymatic and non-enzymatic degradation [58]. This suggests that CPHA-C possesses the potential to undergo natural degradation at the site of tissue injury. Also, the slight decrease in pH observed during the degradation of CPHA and CPHA-C suggests the release of acidic byproducts, likely originating from the degradation fragments of chitosan, HEMA, and AA [59]. Chitosan, the major component of the CPHA hydrogel system, is a weak cationic polymer whose degradation produces weakly acidic glucosamine and acetic acid. Furthermore, the lower pH environment created by CPHA and CPHA-C degradation products could influence the biological outcome and effectiveness of tenocyte function despite the neutralization by physiological buffers [60].

Both CPHA and CPHA-C demonstrated appreciable cytocompatibility as evidenced by the adherence and survivability of tenocytes, suggesting the absence of cytotoxicity. Also, the direct contact assay revealed significant viability, suggesting the absence of any toxic degradation products which promotes the adhesion, proliferation, and survival of tenocytes [61]. Additionally, the tenocytes exhibited appreciable infiltration, migration and penetration into the hydrogel interstices, which occurred at a greater magnitude in CPHA-C. The increased penetration of tenocytes in the CPHA-C hydrogel has been correlated with collagen content and porosity. The collagen forms a fibrillar interconnected network that naturally increases pore size and enhances internal surface area, providing a native tendon matrix-like environment for the tenocytes [62]. Additionally, collagen presents abundant binding sites for integrins, thereby improving cell adhesion and facilitating deeper cellular infiltration [63]. Translationally, the CPHA-C hydrogel is customizable by varying the collagen content to impart porosity, tendon-mimetic features and ideal scaffolding cues [64].

The interaction of tissue engineering hydrogels with immune cells reflects immunocompatibility. The increased viability of both CPHA and CPHA-C hydrogels upon contact with RAW264.7 cells highlights the absence of macrophage activation, suggesting minimal likelihood of immune activation. Additionally, maintaining the oxidative balance associated with hydrogels is especially crucial to support tissue regeneration and wound healing as oxidative stress plays a significant role in implant rejection [65,66]. Also, higher levels of reactive oxygen species (ROS) result in apoptosis, impaired cell proliferation, and reduced tissue regeneration. Hence, hydrogel templates that possess the potential to rescue oxidative stress are critical in alleviating an adverse inflammatory response [67]. In this study, CPHA and CPHA-C hydrogels significantly reduced the reactive oxygen series (ROS) levels in the RAW264.7 macrophages, suggesting their antioxidant potential. Mitochondrial superoxide (Mitosox) reflects cellular oxidative stress and damage. Significantly decreased Mitosox in CPHA-C suggests the role of collagen in providing cellular support and modulating the inflammatory response. Evidently, collagen provides enhanced cellular homeostasis and reduced metabolic strain on the mitochondria, which leads to decreased mitochondrial superoxide production [68]. Similarly, both CPHA and CPHA-C displayed a reduction in lipid peroxidation compared to the cumene control, suggesting their potential to withstand cellular damage related to lipid peroxidation resulting from oxidative stress [69].

The ratio of TGFβ and TLR4 expression levels in RAW264.7 macrophages and tenocytes were utilized to assess the possible inflammatory and regulated immune responses associated with CPHA and CPHA-C. In tendon tissue, TGFβ promotes tissue repair, fibroblast activation and ECM restoration [70], whereas TLR-4 triggers inflammation and immune response [9,10]. The increased level of TLR4 in both cell types upon encountering CPHA and CPHA-C hydrogels reflects the transient inflammatory response which triggers the expression of TGFβ [71,72,73]. Notably, the increased level of mature COL-I inhibits TGFβ signaling as a feedback mechanism [74], which explains the decreased TGFβ expression in both cells upon encountering CPHA-C hydrogels. These observations suggest that the incorporation of collagen in CPHA-C facilitates a more pronounced micro niche for tendon regeneration despite the acute inflammatory milieu elicited by TLR4 signaling.

TREM1 is intimately associated with aggravated inflammation, responsible for the development of RCTI [9,10]. TREM-1-like transcript-1 (TLT1)-derived LR12 peptide has been identified as a potent inhibitor of TREM1, and LR12-directed TREM1 inhibition has been successfully attempted in several inflammatory disorders [75]. LR12-loaded CPHA and CPHA-C hydrogels displayed sustained release over the course of 9 days. Macrophages display transient inflammation upon acute exposure to biomaterials, which is necessary for activating healing responses [76,77]. Logically, CPHA and CPHA-C hydrogels exhibited increased TREM1 expression to activate inflammation-driven healing; however, this warrants further validation. Moreover, the LR12-loaded CPHA hydrogel displayed appreciable TREM1 inhibition in LPS-challenged RAW264.7 macrophages, whereas the effect was minimal for CPHA-C. Reasonably, LR12 possesses strong affinity to the otherwise hydrophilic CPHA-C hydrogels owing to the presence of hydrophilic amino acids. Importantly, LR12 activity has a very short half-life of 2.25 min [13,78,79]. Hence, the retention of the TREM1-inhibitory activity of LR12 in hydrogels is challenging due to its hydrophilicity. Despite the promising release profile of both hydrogels, the maintenance of LR12 activity warrants further optimization in terms of dose and retention time. Additional engineering strategies including encapsulation of LR12 to protect its activity or using lentiviral approaches for sustained secretion using these hydrogels could be promising; however, this warrants further investigation.

Limitations of study: Despite the promising findings, this study is limited by the lack of scalability to control pore formation and pore consistency by freeze-drying and the lack of validation with primary macrophages (immortalized cell line such as RAW267.4 may not replicate the typical inflammatory milieu in vitro); the unavailability of specific antibodies for LR12 prevented us from quantifying the release by ELISA/WB. Moreover, as the tendon is a load-bearing tissue, the lack of mechanical characterization of CPHA and CPHA-C hydrogels warrants further investigation; the increased TLR4 expression, minimal retention of LR12 activity, and increased degradation rate associated with collagen warrant further optimization in future formulations of hydrogels. Additionally, this study lacks in vivo validation assessing the performance of CPHA hydrogels, limiting the extrapolation of their translational potential to the clinical context.

Overall, this study demonstrates that both CPHA and CPHA-C hydrogels offer promise as biocompatible, structurally intact, and cell-supportive scaffolds for tenocyte delivery in tendon regeneration methods. The incorporation of collagen in CPHA-C enhanced porosity, tendon-mimetic features, hydrophilicity, and cell infiltration while modulating oxidative and inflammatory responses. These findings contribute to the utilization of both CPHA and CPHA-C hydrogel systems for tendon repair and highlight their immunocompatibility, which allows for better mimicking of the ideal biologic tendon environment. Additionally, the findings from this study can pave multiple future avenues in RCTI management including localized TREM1 inhibition, Collagen-I replenishment, immunomodulation, and tendon regeneration in the clinical context.

## 3. Conclusions

CPHA and CPHA-C hydrogels displayed physiochemical and biological properties, progressive biodegradation, water kinetics, immuno/biocompatibility, an amphiphilic nature, as evident by contact angle analysis (~70 for CPHA and CPHA-C), and surface porosity (28.65 ± 6.85 and 41.56 ± 18.00, respectively, for CPHA and CPHA-C). Both hydrogels supported the growth and proliferation of tenocytes (>90% viability) within the networks and masked oxidative damage, eliciting minimal immune response. Notably, the TGFβ/TLR4 ratio was elevated in the CPHA hydrogel (0.24), suggesting an anti-inflammatory profile, while the reduced ratio in CPHA-C (−0.52) was likely influenced by the presence of collagen. Additionally, both hydrogels displayed an excellent LR12 release profile; however, CPHA was effective in terms of TREM1 inhibition. Collectively, these findings reflect the promising potential of CPHA and CPHA-C hydrogels to serve as immunocompatible scaffold systems for tendon regeneration.

## 4. Materials and Methods

### 4.1. Materials

Chemicals and reagents utilized for the preparation and characterization of hydrogels were of synthetical or analytical grade. The details of the key chemicals, reagents and kits are as follows: chitosan (Catalogue# 448877), L-Ascorbic Acid (Catalogue# A7506), Ammonium persulfate (APS) (Catalogue# A3678), Polyvinyl Alcohol (PVA) (Catalogue# 363138), glacial acetic acid (Catalogue# 695092), dimethyl sulfoxide (DMSO) (Catalogue# D2650) and acrylic acid (AA) (stabilized with hydroquinone monomethyl ether for synthesis) (Catalogue# 8001811000) were purchased from Sigma-Aldrich, St. Louis, MO, USA. Bovine collagen (Catalogue# 5162-1GM) was obtained from Advanced BioMatrix (ABM), Carlsbad, CA, USA. Hydroxyethyl Methacrylate (HEMA) (Catalogue# B24260), MTT (Catalogue# M6494), Triton x100 (Catalogue# 85111), Pierce Bicinchoninic Acid (BCA), the Protein Assay Kit (Catalogue# 23225), and phosphate-buffered saline (PBS) (Catalogue# BR0014G) were obtained from Thermo Fisher Scientific, Waltham, MA, USA. Cell staining kits including rhodamine–phalloidin (Catalogue# R415), MitoSOX (Catalogue# M36008), lipid peroxidation (Catalogue# C10445), and cell ROX (Catalogue# C10492) were purchased from Invitrogen, Waltham, MA, USA.

### 4.2. Methodology

#### 4.2.1. Synthesis of Collagen-I-Containing Hydrogel Using Chitosan, PVA, and HEMA-AA Redox System

The hydrogels were prepared using solvent casting coupled with slow evaporation in an aqueous phase where chitosan and PVA formed the major components. Briefly, 20 g of 3% (*w*/*v*) chitosan (in 1% glacial acetic acid) was blended with 4 mL 5% PVA (*w*/*v*) and warmed under constant stirring to form a homogeneous mixture. The weight ratio of chitosan and PVA was 6:2. Subsequently, HEMA (2 mL) and acrylic acid (AA) (1 mL) were added to the mixture and stirred, followed by the addition of redox initiators ascorbic acid (5% *w*/*v*) and APS (5% *w*/*v*). The resulting homogenous mixture was cast and oven-dried to form the sheet of chitosan–PVA interpenetrated with AA-HEMA (CPHA). To prepare collagen-incorporated hydrogels (CPHA-C), 4 mL of bovine collagen-1 (1 mg/mL) was added to the CPHA mixture prior to casting (Figure 1). The temperature for the entire reaction was maintained below 42 °C to prevent the denaturation of Collagen-I and preserve biological function. Following synthesis (after overnight casting and evaporation), both CPHA and CPHA-C hydrogels were rehydrated and washed in sterile distilled water to remove unreacted chemicals, freeze-dried to induce porosity, UV-sterilized, and stored aseptically for further studies.

#### 4.2.2. Physiochemical Characterizations

The physiochemical characterizations of CPHA and CPHA-C hydrogels were performed using attenuated total reflectance infrared (ATR-IR) analysis to identify the surface functional groups, scanning electron microscopy (SEM) for surface morphometry and pore length (n = 9), water contact angle analysis (n = 5; water swollen hydrogels of 1 cm × 5 cm dimension) for surface hydrophilicity using Wilhelmy’s method, swelling studies in distilled water for water uptake kinetics (n = 5), and differential scanning calorimetry (DSC) for water transition states, following protocols established in our previous publication [26].

#### 4.2.3. Biodegradation

The degradation profiles of CPHA and CPHA-C hydrogels (*n* = 4) were determined in PBS (pH = 7.34) at 37 °C by assessing the weight loss and alterations in pH on day 2, day 5 and day 10, as reported previously [26].

#### 4.2.4. Biological Evaluation

##### Tenocyte Culture and Maintenance

Tenocytes were isolated and characterized from the shoulder tendon of Yucatan mini-swine, as reported previously [28], and were maintained in DMEM with 20% FBS (fetal bovine serum) under standard cell culture conditions. Cells growing to ~80% confluence were sub-cultured and passages 2–5 were used for the studies.

##### Interaction of Tenocytes in CPHA and CPHA-C Hydrogels

CPHA and CPHA-C hydrogels (1 cm diameter) were preconditioned in DMEM (with 20% FBS) at 37 °C for 48 h. The preconditioned media was used to culture tenocytes for 24 h and viability was assessed using the MTT assay (n = 8) following our previous report [29]. The effect of tenocyte viability upon interaction with CPHA and CPHA-C hydrogels was determined via direct contact assay [26]. Spreading and infiltration of tenocytes on CPHA and CPHA-C hydrogels were determined by rhodamine staining and z-stack analysis using a Leica Thunder microscope (Wetzlar, Germany) [26].

#### 4.2.5. Immuno-Protective Effects of CPHA and CPHA-C Hydrogels

##### Culture and Maintenance of RAW264.7 Macrophages

The mouse macrophage cell line RAW 264.7 (ATCC-TIB-71) was maintained in high-glucose DMEM with 10% FBS (Cat# 30-2020, ATCC) under standard culture conditions (5% CO_2_, 37 °C, and antibiotics). Upon ~80% confluency, the cells were sub-cultured using a cell scraper by replenishing two-thirds of fresh media [30]. Cells of passages 3–10 were used for the studies. CPHA and CPHA-C hydrogels were preconditioned in DMEM with 10% FBS and were placed on top of a sub-confluent monolayer of Raw264.7 cells. The following experiments were performed. Macrophage activation was assessed using a direct contact assay, as described above.

##### Oxidative Stress

Hydrogel-treated Raw264.7 cells (n = 16) were incubated post treatment with CellROX Reagent (Invitrogen, Waltham, MA, USA) (5 μM) for 30 min at 37 °C, washed with serum-free media and imaged under a fluorescence microscope using a blue filter. The MFI was quantified as mentioned above and the results were expressed as log_2_ fold change (FC) normalized to the control [4].

##### Mitochondrial Superoxide

The status of mitochondrial superoxide upon contact with CPHA and CPHA-C hydrogels in the RAW 264.7 cells (n = 16) was assessed using MitoSOX Red (M36008, Invitrogen, Waltham, MA, USA) [80]. After the hydrogel treatments, the RAW 264.7 cells were incubated with 5 µM MitoSOX Red reagent for 10 min and imaged under a fluorescent microscope (Leica Thunder, Wetzlar, Germany). The MFI was quantified from the images, normalized to that of untreated controls and represented as log_2_ FC.

##### Lipid Peroxidation Assay

Lipid peroxidation was assessed after treatment using the Image-iT Lipid Peroxidation kit (C10445, Life technologies, Carlsbad, CA, USA), strictly following the manufacturer’s recommendations [81]. Post treatment, cells (n = 16) were washed with serum-free DMEM (Waltham, MA, USA), incubated with 10 mM 200 µL Image-iT^®^ Lipid Peroxidation Sensor (Waltham, MA, USA) for 30 min at 37 °C, and washed again with serum-free DMEM, and fluorescence images were taken at 590 nm and 510 nm using a fluorescence microscope (Leica Thunder, Wetzlar, Germany). The MFI was quantified using ImageJ software (version 1.46r) and the ratio of the MFI at 590 nm to 510 nm was calculated to assess the extent of lipid peroxidation. RAW 264.7 cells treated with 100 µM cumene hydroperoxide (CuP) served as the positive control.

#### 4.2.6. Expression of TLR4 and TGFβ in Tenocytes and RAW264.7 Macrophages

Both tenocytes and RAW264.7 were grown on the surface of and in contact with CPHA and CPHA-C hydrogels (n = 9). Expression levels of the pro-inflammatory receptor TLR4 and the pro-healing mediator TGFβ were determined using immunostaining following our previous protocols [80]. Primary antibodies against TLR4 and TGFβ at a dilution of 1:400 were used for binding. Fluorochrome-conjugated secondary antibodies (1:500) were used for detection. Nuclei were stained with 4′,6-diamidino-2-phenylindole (DAPI) and imaged using a fluorescent microscope (Leica Thunder, Wetzlar, Germany). The MFI was quantified and normalized to the number of cells to determine the log_2_ FC.

#### 4.2.7. LR12 Release and TREM1 Inhibition

Freeze-dried CPHA and CPHA-C hydrogels (1 cm discs) were loaded with LR12 (0.3 mg/mL/disc in PBS), allowed to soak at 37 °C for 2 h, and incubated in 10 mL PBS (pH 7.40) at 37 °C for 10 days. The release profile was determined by replacing 1 mL of fresh PBS at 24 h intervals. The LR12 peptide released was quantified by using the standard BCA assay [26]. To assess TREM1 inhibition, the LR12-loaded hydrogels were placed on top of a monolayer of LPS-challenged RAW264.7 macrophages, allowing for TREM1 release over 24 h, and the expression level of TREM1 was determined by immunostaining as mentioned above.

#### 4.2.8. Statistical Analysis

Results were expressed as the mean ± SD. Nuclei were counted using ImageJ software with ‘Analyze particle’ mode for the cell staining experiments and were normalized to the MFI values of corresponding images (a minimum of 5 images) to calculate MFI/cells. Average MFI/cell was utilized to normalize MFI/cell in experimental groups. These results were expressed as the log_2_ FC with respect to the control, where the control value represents zero, as represented in the quantification graphs, values above zero denote upregulation and values below zero indicate downregulation. The images that displayed background fluorescence and outliers were exempted from the analysis. Statistical significance was assessed by one-way ANOVA following the two-stage linear step-up procedure of Brown–Forsythe, Bartlett and Tukey, employing GraphPad Prism software (version 11.0.0). An unpaired *t*-test was used to compare CPHA and CPHA-C hydrogels. Values with *p* < 0.05 were considered significant when comparing between the experimental groups.

## Figures and Tables

**Table 1:** Properties of CPHA and CPHA-C hydrogels.

Parameters	CHPA	CHPA-C	*p* Value
Pore length	28.65 ± 6.85	41.56 ± 18.00	0.0696
Advancing contact angle (°) (n = 5)	71.49 ± 2.91°	68.81 ± 2.25°	0.7751
Receding contact angle (°) (n = 5)	73.04 ± 2.12°	68.84 ± 2.67°	
Swelling (%) (n = 5)	128.40 ± 15.11	119 ± 6.73	0.2900
Equilibrium water content (%) (n = 5)	56.17 ± 6.53	56.04 ± 2.91	0.7308
Total water absorption sites (TWASs) (n = 5)	9.2375 × 10^20^	9.8729 × 10^20^	0.6905
Swelling constant (k) (n = 5)	0.1379	0.2293	0.285
Diffusional exponent (n) (n = 5)	0.344	0.3385	0.1849
Enthalpy of melting endotherm (J/g)	134.2 J/g	145.4 J/g	n/a
Freezing water content (Wf)	39.58%	43.53%	n/a
Non-freezing bound water (Wnb)	17.59%	12.51%	n/a
Tenocyte viability (MTT assay) (%)	110.11 ± 6.51	91.20 ± 8.73	0.0001

## Data Availability

All data and materials are available on request from the corresponding author. The data is not publicly available due to ongoing research using part of the data.

## Abbreviations

ATR-IR	Attenuated total reflectance infrared
BCA	Bicinchoninic acid
CPHA	Chitosan–PVA interpenetrated HEMA crosslinked with AA
CPHA-C	Chitosan–PVA interpenetrated HEMA crosslinked with AA containing collagen
DSC	Differential scanning calorimetry
HEMA	Hydroxyethyl methacrylate
PVA	Polyvinyl alcohol
RTCI	Rotator cuff tendon injury
SEM	Scanning electron microscopy
TLR4	Toll-like receptor-4
TREM1	Triggering receptors expressed on myeloid cells-1
TWAS	Total water absorption site
